# Uncovering the Anti-Lung-Cancer Mechanisms of the Herbal Drug FDY2004 by Network Pharmacology

**DOI:** 10.1155/2021/6644018

**Published:** 2021-02-04

**Authors:** Ho-Sung Lee, In-Hee Lee, Kyungrae Kang, Sang-In Park, Tae-Wook Kwon, Dae-Yeon Lee

**Affiliations:** ^1^The Fore, 87 Ogeum-ro, Songpa-gu, Seoul 05542, Republic of Korea; ^2^Forest Hospital, 129 Ogeum-ro, Songpa-gu, Seoul 05549, Republic of Korea; ^3^Forestheal Hospital, 173 Ogeum-ro, Songpa-gu, Seoul 05641, Republic of Korea

## Abstract

With growing evidence on the therapeutic efficacy and safety of herbal drugs, there has been a substantial increase in their application in the lung cancer treatment. Meanwhile, their action mechanisms at the system level have not been comprehensively uncovered. To this end, we employed a network pharmacology methodology to elucidate the systematic action mechanisms of FDY2004, an anticancer herbal drug composed of Moutan Radicis Cortex, Persicae Semen, and Rhei Radix et Rhizoma, in lung cancer treatment. By evaluating the pharmacokinetic properties of the chemical compounds present in FDY2004 using herbal medicine-associated databases, we identified its 29 active chemical components interacting with 141 lung cancer-associated therapeutic targets in humans. The functional enrichment analysis of the lung cancer-related targets of FDY2004 revealed the enriched Gene Ontology terms, involving the regulation of cell proliferation and growth, cell survival and death, and oxidative stress responses. Moreover, we identified key FDY2004-targeted oncogenic and tumor-suppressive pathways associated with lung cancer, including the phosphatidylinositol 3-kinase-Akt, mitogen-activated protein kinase, tumor necrosis factor, Ras, focal adhesion, and hypoxia-inducible factor-1 signaling pathways. Overall, our study provides novel evidence and basis for research on the comprehensive anticancer mechanisms of herbal medicines in lung cancer treatment.

## 1. Introduction

Despite the advances in anticancer therapies, lung cancer (LC) remains the most common reason for cancer mortality, which accounts for 18.4% of global cancer deaths [1]. Accumulating evidence and increasing understanding of LC pathology have led to the development of effective anticancer therapies such as chemotherapy, targeted therapy, and cancer immunotherapy that can prolong the survival of patients with LC; however, these therapies may frequently and inevitably accompany therapeutic resistance and toxic adverse effects [2, 3]. Therefore, there has been a substantial increase in the application of herbal drugs in cancer treatment owing to their potent anticancer effects and less toxicities [4–6]. It has been shown that the administration of herbal drugs can enhance the efficacy and attenuate the adverse effects of anticancer therapies, alleviate cancer symptoms, and improve the survival and clinical outcomes of patients with cancer [6–8].

FDY2004 is a herbal drug composed of three herbal medicines, namely, Moutan Radicis Cortex (MRC), Persicae Semen (PS), and Rhei Radix et Rhizoma (RRR) (Supplementary Table S1) [9]. This herbal drug may exert potent antiproliferative effect against LC cells (Supplementary Table S1) [9]; however, its system-level anticancer mechanisms in LC treatment remain to be elucidated.

With advances in scientific and analytical technologies, various convergence research methodologies such as network pharmacology have emerged [5, 10–12]. They have been used to investigate complex multiple compound-multiple target pharmacological mechanisms of herbal drugs [5, 10–12]. Network pharmacology is used to explore the mechanisms underlying various diseases and action mechanisms of therapeutic herbal drugs [5, 10–12]. It involves the interactions among biological elements, such as genes, proteins, and metabolites, and integrates pharmacology, medicine, and network biology [5, 10–12]. This research strategy has been proven beneficial in understanding the multiple compound-multiple target mechanisms of herbal drugs via the following: (i) investigation of their active chemical components and disease-associated therapeutic targets and (ii) analysis of their therapeutic functions to uncover the polypharmacological mechanisms of herbal medicines [5, 10–12]. By employing a network pharmacology methodology, we uncovered the anti-LC mechanisms of FDY2004.

## 2. Materials and Methods

### 2.1. Investigation of the Active Chemical Components of FDY2004

We retrieved the chemical components of FDY2004 from the Traditional Chinese Medicine Systems Pharmacology (TCMSP) and Anticancer Herbs Database of Systems Pharmacology (CancerHSP) databases [13, 14]. We then used the pharmacokinetic characteristics obtained from the aforementioned databases [13, 14], including oral bioavailability, Caco-2 permeability, and drug-likeness, to determine the active chemical components of FDY2004. Oral bioavailability is a measure of an orally administered drug's capacity to be transported to the general circulation and sites of drug action [13, 15]. Chemical compounds whose oral bioavailability is higher than 30% are regarded to possess effective absorption abilities [13, 15]. Caco-2 permeability is an indicator of the diffusion ability of a chemical compound in the intestinal epithelium, assessed using Caco-2 human intestinal cells [13]. Compounds with a Caco-2 permeability of ≥−0.4 are considered to have effective permeability in the intestines [16, 17]. Drug-likeness is an index used to investigate the druggability of a chemical component with respect to its biochemical and physical properties using Tanimoto coefficients [13, 18]. The average drug-likeness of available drugs is 0.18; therefore, chemical compounds whose drug-likeness is higher than this average value are regarded to have druggable potential in network pharmacology analysis [13, 18]. Consequently, in this study, chemical components that meet the following criteria were determined to be bioactive: oral bioavailability ≥ 30%, drug-likeness ≥ 0.18, and Caco-2 permeability ≥ −0.4 [11, 13].

### 2.2. Identification of the Targets of Active Chemical Components

We retrieved the simplified molecular-input line-entry system (SMILES) notation for individual chemical components from the PubChem database [19]. By importing the SMILES information into diverse *in silico* tools, involving the SwissTargetPrediction [20], Search Tool for Interactions of Chemicals 5 [21], PharmMapper [22], and Similarity Ensemble Approach [23], the human targets of FDY2004 were obtained. The LC-associated human targets were searched using “Lung Neoplasms” (Medical Subject Headings Unique ID: D008175) as a keyword in the following comprehensive genomic databases: Comparative Toxicogenomics Database [24], Therapeutic Target Database [25], Human Genome Epidemiology Navigator [26], GeneCards [27], DisGeNET [28], DrugBank [29], Online Mendelian Inheritance in Man [30], and Pharmacogenomics Knowledge Base [31].

### 2.3. Construction of Herbal Drug-Associated Networks

Herbal medicine-active chemical component (H-C), active chemical component-target (C-T), and target-pathway (T-P) interaction networks were built by connecting the herbal components of FDY2004 with their active chemical components, the components with their interacting targets, and the targets with their relevant enriched pathways, respectively. A protein-protein interaction (PPI) network was built based on the interaction data for the targets obtained from the STRING database (interaction confidence score ≥ 0.7) [32]. Network visualization and analysis were conducted using Cytoscape [33]. In the network pharmacology analysis, individual constituents relevant to a herbal drug, including its herbal medicines, chemical components, targets, and pathways, are represented as nodes [34]. Their interactions are represented as links (or edges) [34]. The degree of nodes is defined as the number of their links, and nodes with a relatively high degree are called hubs [34].

### 2.4. Survival Analysis

The correlation between the expression status of FDY2004 targets and the survival of patients with LC was analyzed using Kaplan–Meier plotter [35].

### 2.5. Functional Enrichment Analysis

Functional enrichment of Gene Ontology (GO) terms and pathways for “*Homo sapiens*” by FDY2004 targets was analyzed using g:Profiler [36] and Kyoto Encyclopedia of Genes and Genomes [37].

### 2.6. Molecular Docking Analysis

We obtained the structures of the chemical components and their targets from PubChem [19] and RCSB Protein Data Bank [38], respectively. Then, we imported the collected structural information into Autodock Vina [39] and analyzed the docking scores of individual chemical component-target pairs. As reported previously, we considered that a chemical component-target pair might have a high binding affinity if it has a docking score of less than −5.0 [40, 41].

## 3. Results

### 3.1. Active Chemical Components of FDY2004

From the TCMSP and CancerHSP [13, 14], we obtained detailed information on the chemical components of FDY2004 (Supplementary Table S2). The chemical components that satisfied the following criteria were considered bioactive: oral bioavailability ≥ 30%, drug-likeness ≥ 0.18, and Caco-2 permeability ≥ −0.4 [11, 13]. We also considered numerous compounds as active compounds because of their high amounts and potent activity, although they did not meet the aforementioned criteria [42–56]. Thus, 35 bioactive chemical compounds of FDY2004 were identified (Supplementary Table S3).

### 3.2. Targets of the Active Chemical Components of FDY2004

We obtained 212 human molecular targets for the 29 bioactive chemical components of FDY2004 (see Materials and Methods) (Supplementary Table S4). The information on the LC-associated human genes and proteins was retrieved from various genomic databases (see Materials and Methods), and 141 of all the 212 FDY2004 targets were considered LC-related targets.

### 3.3. Network Pharmacological Identification of the Action Mechanisms of FDY2004

**Figure 1:**
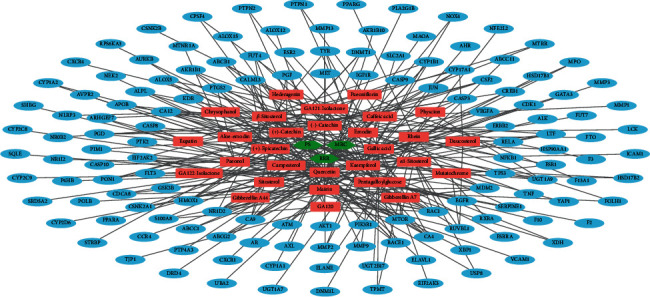
The herbal medicine-active chemical component-target network of FDY2004. Green hexagon nodes, herbal medicines; red rectangle nodes, active chemical compounds; blue eclipse nodes, lung cancer-related targets.

By integrating the comprehensive data of FDY2004, including its herbal and chemical components and their LC-related targets, we built a herbal medicine-active chemical component-target (H-C-T) network representing the polypharmacological features of the herbal drug (Figure 1). The network consisted of 173 nodes (3 herbal medicines, 29 bioactive chemical components, and 141 targets) and 304 links (Figure 1 and Supplementary Table S4). In this network, quercetin, kaempferol, gallic acid, emodin, campesterol, (+)-epicatechin, (+)-catechin, and (−)-catechin had the highest number of interacting targets (Figure 1), demonstrating that they may be the key pharmacological compounds underlying the anti-LC effects of FDY2004. It is noteworthy that 96 of the 141 LC-related genes/proteins were common therapeutic targets of two or more bioactive chemical components of FDY2004 (Figure 1), implying the polypharmacological and coordinated action mechanisms of FDY2004.

**Figure 2:**
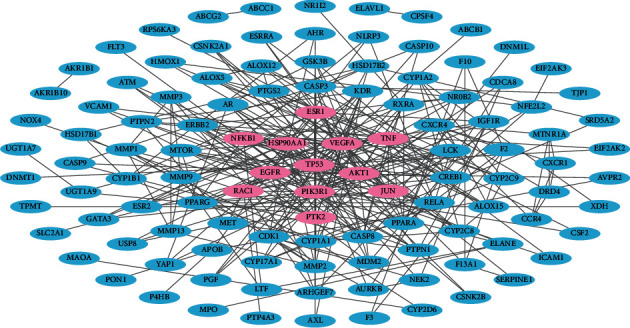
The protein-protein interaction network for lung cancer-related targets of FDY2004. Pink nodes, hub targets.

**Figure 3:**
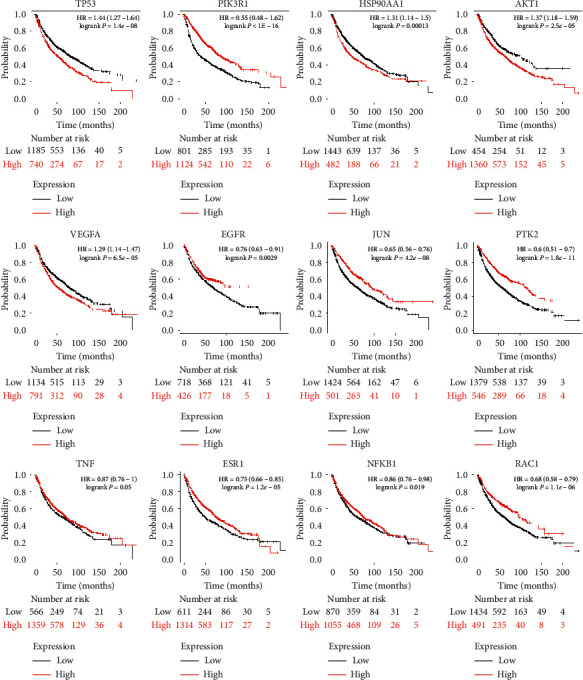
Survival analysis of lung cancer-related hub targets of FDY2004. Kaplan–Meier curves for overall survival of the patients with lung cancer with respect to the expression of the indicated targets.

To understand the interactions among the LC-related targets of FDY2004, a PPI network with 114 nodes and 304 edges was generated, where the targets served as nodes and their interactions represented edges (Figure 2). We then searched for nodes with a relatively high degree (i.e., hubs) [57, 58]. They are reported to have key roles in the pharmacological activities of drugs and serve as potential therapeutic targets [57, 58]. As reported previously, hubs were defined as nodes with a degree higher than or equal to twice the average degree of all nodes in a PPI network [11]. The nodes TP53, PIK3R1, HSP90AA1, AKT1, VEGFA, EGFR, JUN, PTK2, TNF, ESR1, NFKB1, and RAC1 were identified as hubs with high degree (Figure 2), demonstrating that these targets may be important for the exertion of anti-LC pharmacological effects of FDY2004. The expression status of these hub targets was further shown to be significantly related to the survival of patients with LC (Figure 3), implying their potential clinical significance and prognostic role.

### 3.4. Functional Enrichment Analysis of FDY2004-Associated Targets and Pathways

To explore the molecular mechanisms of FDY004 in LC treatment based on the biological functions of its targets, we carried out the GO enrichment analysis. The GO terms involved in the various biological functions, including cell proliferation and growth, cell survival and death, and oxidative stress responses, were enriched by the LC-related targets of FDY2004 (Supplementary Figure S1), indicating the anticancer molecular characteristics of its pharmacological activity.

**Figure 4:**
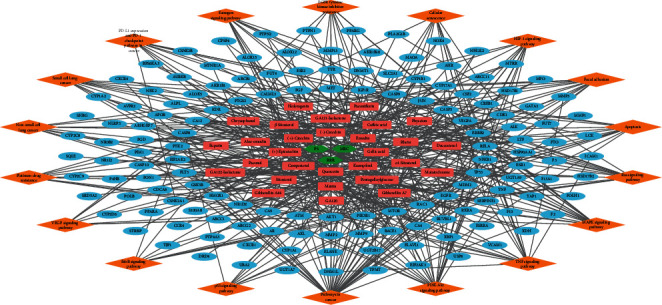
The herb-compound-target-pathway network of FDY2004. Green hexagon nodes, herbal medicines; red rectangle nodes, active chemical compounds; blue eclipse nodes, lung cancer-related targets; orange diamond nodes, signaling pathways.

To investigate the pathway-level pharmacological properties of FDY2004 against LC, we conducted the pathway enrichment analysis (Figure 4 and Supplementary Figure S1). The following signaling pathways were found to be enriched by the LC-associated targets of FDY2004: “Pathways in cancer,” “PI3K-Akt signaling pathway,” “MAPK signaling pathway,” “TNF signaling pathway,” “Ras signaling pathway,” “Apoptosis,” “Focal adhesion,” “HIF-1 signaling pathway,” “Cellular senescence,” “EGFR tyrosine kinase inhibitor resistance,” “Estrogen signaling pathway,” “PD-L1 expression and PD-1 checkpoint pathway in cancer,” “Small cell lung cancer,” “Non-small cell lung cancer,” “Platinum drug resistance,” “ErbB signaling pathway,” “p53 signaling pathway,” and “VEGF signaling pathway” (Figure 4 and Supplementary Figure S1).

Together, the results suggest the system-level mechanisms of FDY2004 against LC from the molecular- and pathway perspectives.

### 3.5. Molecular Docking of the FDY2004 Targets

**Figure 5:**
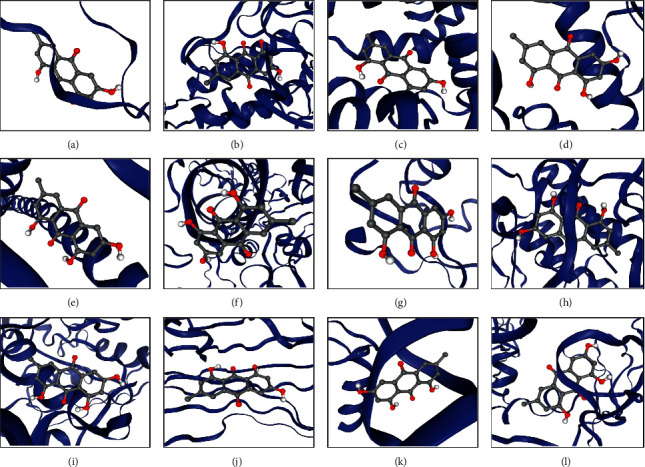
Molecular docking analysis for the active chemical compounds of FDY2004 and the targets. The analysis results of quercetin and the hub targets are shown as representatives. (a) Quercetin-AKT1 (score = −6.4). (b) Quercetin-EGFR (score = −7.9). (c) Quercetin-ESR1 (score = −6.5). (d) Quercetin-HSP90AA1 (score = −6.1). (e) Quercetin-JUN (score = −5.4). (f) Quercetin-NFKB1 (score = −6.6). (g) Quercetin-PI3KR1 (score = −6.3). (h) Quercetin-PTK2 (score = −6.1). (i) Quercetin-RAC1 (score = −9.3). (j) Quercetin-TNF (score = −6.5). (k) Quercetin-TP53 (score = −7.0). (l) Quercetin-VEGFA (score = −7.2).

To investigate the binding activities of compound-target interactions for FDY2004, we analyzed their molecular docking affinities (see Materials and Methods). In the docking analysis, 95.19% of the active compound-target pairs presented docking scores of ≤−5.0, implying the potential pharmacological binding activities of the herbal drug (Figure 5 and Supplementary Table S5).

## 4. Discussion

Although there has been increasing use of herbal drugs in LC treatment, their system-level anticancer mechanisms have not been comprehensively understood. Here, we employed a network pharmacological approach to uncover the therapeutic mechanisms of FDY2004 [9] in LC treatment from a system-level view. The network pharmacological investigation of FDY2004 revealed 29 active chemical components that interact with 141 lung cancer-associated therapeutic targets, mediating the anti-LC effects of the herbal drug. The GO enrichment analysis of the FDY2004 targets revealed the molecular action mechanisms of FDY2004, involving the regulation of cell proliferation and growth, cell survival and death, and oxidative stress responses. Furthermore, the key FDY2004-targeted oncogenic and tumor-suppressive pathways implicated in LC development and progression were the phosphatidylinositol 3-kinase (PI3K)-Akt, mitogen-activated protein kinase (MAPK), tumor necrosis factor (TNF), Ras, focal adhesion, and hypoxia-inducible factor (HIF)-1 signaling pathways.

The LC-related hub targets of FDY2004 were found to be closely associated with LC pathology and play a role as prognostic indicators for the survival and therapeutic sensitivity of patients with LC. The tumor suppressor *TP53* is one of the most frequently mutated and malfunctioned genes in the pathological process of LC, and its genetic and functional status may serve as a predictor for the risk, survival, and therapeutic outcomes of LC [59–63]. *PIK3R1* is involved in the regulation of LC cell growth [64]. The upregulation of *HSP90AA1* correlates with the occurrence, progression, and clinical outcomes of LC, and its inhibition can repress the proliferation, survival, and metastasis of LC cells [65, 66]. The abnormal regulation of Akt1 (encoded by *AKT1*) and TNF-*α* (encoded by *TNF*) may enhance the growth, survival, proliferation, metastasis, epithelial-to-mesenchymal transition (EMT), and stemness capacity of LC cells, and they are potential targets that can alleviate chemotherapy and radiotherapy resistance [67–76]. Clinical studies have also reported that *AKT1* and *TNF* may be prognostic determinants for patients' survival and treatment outcomes with LC [73, 77–80]. Vascular endothelial growth factor (VEGF)-A (encoded by *VEGFA*) enhances the metastasis and angiogenesis of LC cells and thereby contribute to the progression of LC, and its activation profile is related to a poor clinical prognosis and the survival of patients with LC [81–86]. Dysregulated expression of *EGFR* and its encoded receptor tyrosine kinase activity may lead to the induction of various cancerous cellular processes underlying the LC pathology, making it a key target of widely used antitumor agents against LC in clinical settings [87–89]. c-Jun (encoded by *JUN*) functions as a modulator of the growth, proliferation, and apoptosis of LC cells as well as a mediator of the pharmacological effects of cytotoxic drugs [90–92]. Pharmacological modulation of focal adhesion kinase (FAK; encoded by *PTK2*) and Ras-related C3 botulinum toxin substrate 1 (RAC1; encoded by *RAC1*) reduces the proliferation, migration, invasion, EMT, motility, angiogenesis, and stemness activity of LC cells, and this reverses chemotherapy and radiotherapy resistance [93–103]. The expression of estrogen receptor (ER)-*α* (encoded by *ESR1*) might be correlated with the survival and prognosis of patients with LC, and previous studies have reported its role as a therapeutic target in LC treatment [104, 105]. The polymorphisms of *NFKB1* are associated with the risk of LC occurrence [106].

The signaling pathways targeted by FDY2004 are known to function as crucial regulators of LC development and progression, mediate treatment resistance to anticancer therapies, and play a role as therapeutic targets. The PI3K-Akt, MAPK, Ras, focal adhesion, HIF-1, and erythroblastic leukemia viral oncogene homolog (ErbB) signaling pathways coordinate diverse tumorigenic processes of cancer cells, involving cell proliferation and growth, survival and cell death, anoikis resistance, metastasis, EMT, self-renewal potential and stemness properties, and angiogenesis, of LC cells [101, 102, 107–125]. In addition, aberrant regulations of these signaling pathways may contribute to therapeutic resistance, which can be overcome by genetic and pharmacological interventions of their activities [101, 102, 107–125]. The TNF signaling pathway is a key inflammation mediator involved in the development, progression, metastasis, and recurrence of LC, and the pathway constituents have prognostic significance in the clinical outcome of patients with LC [74–76, 126, 127]. The estrogen pathway and its components may possess carcinogenic properties in LC and act as potential targets [128–131]. The programmed cell death protein 1 (PD-1)/programmed death-ligand 1 (PD-L1) pathway is involved in the regulation of tumor-related immune processes, and it is a key target of cancer immunotherapy, which attempts to suppress immune escape and enhance antitumor immunity for the durable regression of malignant tumors of LC [132–134]. The dysfunction of genes and proteins comprising the p53 pathway, one of the common carcinogenic causes, is associated with various cancerous behaviors of LC cells, such as uncontrolled proliferation, survival, and cell cycle progression [61, 63, 135–143]. The genetic and functional activities of the pathway components might be correlated with the survival and anticancer therapeutic sensitivity of patients with LC [61, 63, 135–137, 139, 141–143]. The VEGF pathway may induce the progression of LC tumors by activating malignant angiogenic, metastatistic, and proliferative programs of cancer cells, and it is the primary pharmacological target of antiangiogenic anticancer drugs [144–146]. Defects in the regulation of important cellular phenotypes such as apoptosis and cellular senescence are the major drivers of the development and progression of LC, and their proper regulation is the key mechanism of anticancer therapeutics [147–152]. Resistance to platinum-based chemotherapeutics and epidermal growth factor receptor (EGFR) tyrosine kinase inhibitors is mediated by diverse oncogenic signaling mechanisms, and co-targeting the resistance-associated pathways may enhance the efficacy of LC treatment [153–157].

The active chemical components of FDY2004 have been reported to act as anticancer compounds in LC. Aloe-emodin induces DNA damage, autophagy, and death of LC cells by regulating reactive oxygen species (ROS) generation and signaling activities of the PI3K/Akt/mammalian target of rapamycin, MAPK, protein kinase C (PKC), and caspase pathways [158–162]. It also functions as a photosensitizer that enhances irradiation-induced anoikis in LC cells [158–162]. Caffeic acid has been shown to improve the cytotoxicity of chemotherapeutics in LC cells [163]. Catechins may suppress the growth and promote cell cycle arrest of LC cells by inactivating proliferation-inducing oncogenic kinases and cell cycle regulators [164, 165]. Chrysophanol regulates the activation of oxidative stress responses and relevant signaling pathways to reduce the proliferation, migration, invasion, and survival potential of LC cells [166, 167]. Daucosterol disturbs redox homeostasis and cell cycle processes to elicit growth arrest and death of LC cells [168, 169]. Emodin inhibits cell proliferation and migration and promotes EMT, autophagic cell death, and cell cycle arrest coordinated by chemokine, endoplasmic reticulum (ER) stress, ROS, p53, cell cycle, nuclear factor kappa-light-chain-enhancer of activated B cells (NF-*κ*B), tribbles pseudokinase 3, and PKC signaling; it enhances the efficacy of anticancer drugs [159, 170–180]. The proapoptotic and chemosensitizing effects of gallic acid are mediated by the EGFR, PD-L1, ROS, NF-*κ*B, caspase, janus kinase (JAK)-signal transducer and activator (STAT), and mitochondrial pathways [181–188]. Hederagenin exerts cytotoxic effects and further synergizes with chemotherapeutic agents in LC cells [189]. Kaempferol may block the growth, survival, EMT, and migration of LC cells and enhance anti-LC therapies [190–192]. Previous studies have reported the anticancer roles of mairin (betulinic acid) in inducing apoptosis, suppressing proliferation, and reversing drug resistance of LC cells [193–195]. The antiproliferative, antimetastatic, and cell cycle arrest activities of paeoniflorin are mediated by the modulation of the FAS pathways and macrophage activation [196, 197]. Paeonol represses the proliferation and bone metastasis of LC cells and also serves as a radiosensitizer by inhibiting the PI3K/Akt pathway to enhance their apoptosis [198, 199]. Physcion increases the pharmacological sensitivity of LC cells to cytotoxic drugs [200]. Rhein induces apoptosis while suppressing the proliferation of LC cells mediated by the modulation of the calcium, ER stress, and STAT3 pathways [201, 202]. Previous studies have reported the inhibitory roles of quercetin on the growth, survival, metastasis, and chemotherapy and radiotherapy resistance of LC cells via cancer pathways such as Akt, MAPKs, NF-*κ*B, inflammation, and apoptotic caspase signaling [203–209]. *β*-Sitosterol inhibits cancerous autophagic, proliferative, survival, and cell cycle regulatory processes in LC cells [169, 210, 211]. These observations support the pharmacological mechanisms underlying the anti-LC effects of FDY2004.

Overall, our study presents novel and comprehensive insights into and evidence of the anti-LC effects of FDY2004. Further preclinical and clinical studies are warranted to investigate the action mechanisms of FDY2004 and evaluate the pharmacological effects of its combinatorial use with standard anticancer strategies such as chemotherapy, targeted therapy, cancer immunotherapy, and radiotherapy.

## Data Availability

The data used to support the findings of this study are included within the article and Supplementary Materials file.

## Supplementary Materials

Supplementary Materials Supplementary Figure S1: functional enrichment analysis for the lung cancer-related targets of FDY2004. Supplementary Table S1: general information and reports on evidence of biological activities of FDY2004 and its herbal constituents. Supplementary Table S2: chemical components of FDY2004. Supplementary Table S3: active chemical components of FDY2004. Supplementary Table S4: Targets of active chemical components of FDY2004. Supplementary Table S5: docking scores between active chemical components of FDY2004 and the lung cancer-associated targets.
