# Supplementary material for: Uncovering the Anti-Lung-Cancer Mechanisms of the Herbal Drug FDY2004 by Network Pharmacology
Source: Evid Based Complement Alternat Med. 2021 Feb 4;2021:6644018. doi: 10.1155/2021/6644018 (PMC7886515; doi:10.1155/2021/6644018)
Supplement: Supplementary Materials — Supplementary Figure S1: functional enrichment analysis for the lung cancer-related targets of FDY2004. Supplementary Table S1: general information and reports on evidence of biological activities of FDY2004 and its herbal constituents. Supplementary Table S2: chemical components of FDY2004. Supplementary Table S3: active chemical components of FDY2004. Supplementary Table S4: Targets of active chemical components of FDY2004. Supplementary Table S5: docking scores between active chemical components of FDY2004 and the lung cancer-associated targets. [file 6644018.f1.pdf]

Supplementary Materials

Uncovering the anti-lung cancer mechanisms of the herbal drug FDY2004 by network pharmacology

Ho-Sung Lee[1,2], In-Hee Lee[1], Kyungrae Kang[2], Sang-In Park[3],

Tae-Wook Kwon[2], and Dae-Yeon Lee[1,2,]

[1]The Fore, 87 Ogeum-ro, Songpa-gu, Seoul 05542, Republic of Korea.

[2]Forest Hospital, 129 Ogeum-ro, Songpa-gu, Seoul 05549, Republic of Korea.

[3]Forestheal Hospital, 173 Ogeum-ro, Songpa-gu, Seoul 05641, Republic of Korea.

Correspondence should be addressed to Dae-Yeon Lee; foresthrnd@gmail.com

Supplementary Figures

	Category	Term name	Count	Percent [%]	p-value
Gene ontology	Biological process	Cellular response to chemical stimulus	101	2.95	1.93E-39
		Cellular response to chemical stress	27	7.34	1.25E-15
		Cellular response to cytokine stimulus	43	3.76	1.74E-15
		Cellular response to growth factor stimulus	21	2.87	4.45E-04
		Cellular response to radiation	14	7.25	6.53E-07
		Cellular response to stimulus	117	1.50	9.93E-20
		Cytokine-mediated signaling pathway	37	4.57	1.54E-15
		Integrated stress response signaling	4	18.18	4.58E-02
		Lymphocyte activation	19	2.45	1.82E-02
		Lymphocyte differentiation	13	3.46	1.52E-02
		Regulation of angiogenesis	16	3.88	2.69E-04
		Regulation of apoptotic process	63	3.92	2.56E-26
		Regulation of apoptotic signaling pathway	25	6.01	3.09E-12
		Regulation of catabolic process	24	2.31	3.01E-03
		Regulation of catalytic activity	59	2.46	6.18E-14
		Regulation of cell death	67	3.80	1.04E-27
		Regulation of cell growth	15	3.55	2.07E-03
		Regulation of cell migration	37	3.73	1.09E-12
		Regulation of cell motility	38	3.61	1.17E-12
		Regulation of cell population proliferation	55	3.09	3.85E-17
		Regulation of cellular localization	24	2.39	1.62E-03
		Regulation of cellular response to oxidative stress	7	7.37	1.84E-02
		Regulation of cellular response to stress	25	3.19	3.17E-06
		Regulation of cytokine production	23	2.76	2.19E-04
		Regulation of immune response	23	2.26	7.20E-03
		Regulation of immune system process	44	2.67	1.86E-10
		Regulation of inflammatory response	23	5.30	6.66E-10
		Regulation of kinase activity	28	3.04	8.36E-07
		Regulation of leukocyte adhesion to vascular endothelial cell	7	18.42	3.04E-05
		Regulation of leukocyte cell-cell adhesion	19	5.65	2.96E-08
		Regulation of leukocyte differentiation	14	4.76	1.44E-04
		Regulation of metabolic process	95	1.31	6.28E-08
		Regulation of programmed cell death	65	3.98	8.24E-28
		Regulation of response to oxidative stress	7	6.73	3.34E-02
		Regulation of response to stimulus	84	1.91	5.69E-16
		Regulation of response to stress	46	2.98	7.02E-13
		Regulation of signal transduction	71	2.19	2.10E-15
		Regulation of signaling	76	2.05	2.22E-15
		Reponse to chemical	113	2.35	1.82E-37
		Reponse to cytokine	46	3.72	1.18E-16
		Reponse to drug	27	6.44	3.42E-14
		Reponse to growth factor	21	2.77	8.26E-04
		Reponse to radiation	28	6.03	4.45E-14
		Reponse to stimulus	124	1.31	7.20E-17
		Reponse to toxic substance	13	5.20	1.56E-04

	Category	Term name	Count	Percent [%]	p-value
Gene ontology	Cellular components	Cell surface	24	2.64	1.83E-05
		Chromatin	22	1.75	4.57E-02
		Cytoplasm	113	0.96	1.12E-03
		Cytosol	61	1.17	1.28E-02
		Death-inducing signaling complex	3	33.33	9.92E-03
		Endoplasmic reticulum	37	2.01	3.84E-06
		Extracellular matrix	14	2.53	2.01E-02
		Extracellular region	56	1.22	9.27E-03
		Membrane region	12	3.51	2.95E-03
		Nucleoplasm	49	1.22	4.29E-02
		Organelle	127	0.91	2.44E-04
		Plasma membrane	65	1.15	9.60E-03
		Protein-containing complex	63	1.14	1.77E-02
		Transcription regulator complex	13	2.91	9.09E-03
		Vesicle	54	1.34	8.84E-04
	Molecular function	Catalytic activity	96	1.63	3.39E-16
		Drug binding	7	6.86	8.59E-03
		Enzyme binding	55	2.41	3.71E-13
		Hormone binding	6	6.98	3.34E-02
		Kinase activity	23	2.85	2.86E-05
		Kinase binding	22	2.91	3.88E-05
		Ligand-activated transcription factor activity	9	20.45	2.47E-08
		Molecular transducer activity	26	1.76	2.88E-02
		Nuclear receptor activity	9	20.45	2.47E-08
		Phosphatase binding	13	6.63	2.20E-06
		Signaling receptor activity	26	1.76	2.88E-02
		Signaling receptor binding	35	2.09	1.49E-05
		Small molecule binding	49	1.90	1.98E-07
		Steroid hormone receptor activity	9	16.36	2.07E-07
		Transcription factor binding	28	3.97	3.14E-10
Pathway	KEGG	Apoptosis	13	9.56	2.00E-05
		Cellular senescence	11	7.05	3.31E-03
		EGFR tyrosine kinase inhibitor resistance	11	13.92	3.67E-06
		ErbB signaling pathway	8	9.52	4.78E-03
		Estrogen signaling pathway	11	8.03	9.77E-04
		Focal adhesion	13	6.50	1.53E-03
		HIF-1 signaling pathway	12	11.01	1.26E-05
		MAPK signaling pathway	17	5.78	3.57E-04
		Non-small cell lung cancer	9	13.24	1.02E-04
		p53 signaling pathway	8	10.96	1.71E-03
		Pathways in cancer	39	7.37	1.17E-14
		PD-L1 expression and PD-1 checkpoint pathway in cancer	11	12.36	1.29E-05
		PI3K-Akt signaling pathway	22	6.23	3.03E-06
		Platinum drug resistance	9	12.50	1.67E-04
		Ras signaling pathway	14	6.06	1.59E-03
		Small cell lung cancer	10	10.87	1.63E-04
		TNF signaling pathway	17	15.18	1.38E-10
		VEGF signaling pathway	8	13.56	3.40E-04

Supplementary Figure S1. Functional enrichment analysis for the lung cancer-related targets of FDY2004.

Supplementary Tables

Supplementary Table S1. General information and reports on evidence of biological activities of FDY2004 and its herbal constituents

	Herbal medicines			
	Moutan Radicis Cortex	Persicae Semen	Rhei Radix et Rhizoma	FDY2004
Scientific name	*Paeonia suffruticosa* Andrews	*Prunus persica* Batsch	*Rheum palmatum* Linné	-
Vernacular name	*Mudanpi*	*Taoren*	*Dahuang*	-
Species	Paeoniaceae	Rosaceae	Rheum	-
Traditional uses	Treatment for allergic diseases, atherosclerosis, cutaneous diseases, diabetes, infection, and inflammation [1-4]	Treatment for dysmenorrhea, endometriosis, hypermenorrhea, and infertility [5-8]	Treatment for abdominal pain, constipation, fever, and intense and profuse sweating [9, 10]	-
Parts used	Root barks	Dried ripe seed	Dried root and rhizome	-
Collection site	China	Republic of South Africa	China	-
Yield	-	-	-	19.07 ± 2.14 % (w/w) [11]

Biological activities	Analgesic, anti-allergic, anticancer, antidiabetic, anti-inflammatory, anti-oxidative, antipyretic, hepatoprotective, immunomodulatory, and neuroprotective activities [4, 12-14]	Anti-allergic, anticancer, antidiabetic, anti-inflammatory, and anti-oxidative activities [15-20]	Anticancer, anti-fibrotic, anti-inflammatory, anti-oxidative, and gastrointestinal function regulatory activities [21-23]	Anticancer and antioxidant activities [11]

Supplementary Table S2. Chemical components of FDY2004.

Herbal medicines	Chemical compounds	OB	Caco-2	DL
MRC	(+)-Catechin	54.83	-0.03	0.24
MRC	5-[[5-(4-methoxyphenyl)-2-furyl]methylene]barbituric acid {ZINC9320615}	43.44	0.09	0.3
MRC	Caffeic acid	25.76	0.21	0.05
MRC	Kaempferol	41.88	0.26	0.24
MRC	Mairin	55.38	0.73	0.78
MRC	Paeoniflorin	10.22	-1.69	0.79
MRC	Paeoniflorin_qt	68.18	-0.34	0.4
MRC	Paeonol	28.79	0.93	0.04
MRC	Pentagalloylglucose	3.01	-3.08	0.21
MRC	Quercetin	46.43	0.05	0.28
MRC	(1R)-()-Nopinone	57.86	1.23	0.05
MRC	1-(2,3-dihydroxy-4-methoxyphenyl)ethanone	32.96	0.81	0.05

MRC	3-[(2S,3R,4S,5S,6R)-6-[[(2R,3R,4R)-3,4-dihydroxy-4-(hydroxymethyl)oxolan-2-yl]oxymethyl]-3,4,5-trihydroxyoxan-2-yl]oxy-4,5-dihydroxybenzoic acid	5.6	-2.54	0.63
MRC	3-acetyl-4-hydroxybenzoic acid	37.58	-0.04	0.05
MRC	3-Hydroxy-4-methoxyacetophenone	22.32	0.85	0.04
MRC	4-O-Methylpaeoniflorin	25.71	-1.27	0.78
MRC	4-O-Methylpaeoniflorin_qt	67.24	0.15	0.43
MRC	6-Hydroxycoumarin	15.8	0.73	0.05
MRC	6-o-vanillyoxypaeoniflorin	10.91	-1.58	0.54
MRC	6-o-vanillyoxypaeoniflorin_qt 2	17.71	-0.61	0.37
MRC	apiopaeonoside	16.73	-1.79	0.64
MRC	Apocynin	31.71	0.74	0.04
MRC	Benzoyl paeoniflorin	31.14	-1.35	0.54
MRC	Beta-D-Apiose	118.53	-1.46	0.03
MRC	Beta-sitosterol-beta-d-glucoside	19.58	-0.17	0.62
MRC	Beta-sitosterol-beta-d-glucoside_qt	25.32	1.28	0.75

MRC	BOX	31.55	0.54	0.02
MRC	Sitosterol	36.91	1.32	0.75
MRC	Delta 7-stigmastenol	25.32	1.31	0.75
MRC	DEP	52.19	0.72	0.07
MRC	Eugenol	56.24	1.35	0.04
MRC	Galloyl-oxypaeoniflorin	3.2	-2.18	0.39
MRC	Galloyl-oxypaeoniflorin_qt 2	26.21	-0.54	0.44
MRC	Hexanoic acid	73.08	0.8	0.01
MRC	Methyl salicylate	42.55	1.05	0.03
MRC	Mudanoside A	13.39	-1.42	0.29
MRC	Mudanpinoic,acid,a	13.86	0.6	0.65
MRC	Mudanpioside H	6.77	-1.63	0.61
MRC	Mudanpioside H_qt 2	42.36	-0.39	0.37
MRC	octadecyl (E)-3-(3,4-dihydroxyphenyl)prop-2-enoate	3.18	0.98	0.55
MRC	Oleanic acid	8.41	1.14	0.77

MRC	Oleanolic acid	29.02	0.59	0.76
MRC	Oleic acid	33.13	1.17	0.14
MRC	Oxypaeoniflorin	12.98	-1.91	0.78
MRC	Oxypaeoniflorin_qt	19.4	-0.77	0.44
MRC	Paeonidanin	24.64	-1.28	0.78
MRC	Paeonidanin_qt	65.31	-0.09	0.35
MRC	Paeonolide	6.3	-1.42	0.64
MRC	Paeonoside	18.52	-0.86	0.24
MRC	PHB	30.15	0.39	0.03
MRC	Trochol	15.48	0.84	0.78
MRC	Vanillic acid	35.47	0.43	0.04
MRC	WLN: QR CQ DV1	36.49	0.67	0.03
PS	(+)-Epicatechin	48.96	0.02	0.24
PS	Amygdalin	4.42	-1.91	0.61
PS	Campesterol	37.58	1.31	0.71

PS	GA120	84.85	0.38	0.45
PS	GA121-Isolactone	72.7	-0.26	0.54
PS	GA122	64.79	-0.17	0.5
PS	GA122-Isolactone	88.11	-0.18	0.54
PS	Gibberellin A119	76.36	-0.12	0.49
PS	Gibberellin A44	101.61	-0.13	0.54
PS	Gibberellin A7	73.8	-0.18	0.5
PS	Hederagenin	36.91	1.32	0.75
PS	α1-Sitosterol	43.28	1.41	0.78
PS	(2S)-2-phenyl-2-[(2S,3R,4S,5S,6R)-3,4,5-trihydroxy-6-(hydroxymethyl)oxan-2-yl]oxyacetic acid	8.27	-1.22	0.2
PS	(2S,3R,4S,5S,6R)-2-(benzyloxy)-6-methylol-tetrahydropyran-3,4,5-triol	17.14	-0.71	0.14
PS	[(2S,3R,4S,5S,6R)-3,4,5-trihydroxy-6-(hydroxymethyl)oxan-2-yl] (E)-3-(4-hydroxyphenyl)prop-2-enoate	9.8	-1.09	0.26
PS	[2-[(2S,3R,4S,5S,6R)-3,4,5-trihydroxy-6-(hydroxymethyl)oxan-2-yl]oxyphenyl]methyl (E)-3-(3,4-dihydroxyphenyl)prop-2-enoate	8.22	-1.05	0.69

PS	2,3-didehydro GA69	14.28	-0.17	0.5
PS	2,3-didehydro GA77	88.08	-0.67	0.53
PS	2,3-didehydro GA9	17.03	0.37	0.45
PS	24-Methylenecycloartanol	10.4	1.42	0.79
PS	3-feruloylquinic acid	19.31	-1.01	0.36
PS	3-O-p-coumaroylquinic acid	37.63	-1.2	0.29
PS	4a-formyl-7alpha-hydroxy-1-methyl-8-methylidene-4aalpha,4bbeta-gibbane-1alpha,10beta-dicarboxylic acid	88.6	-0.75	0.46
PS	7-dehydroavenasterol	10.03	1.36	0.76
PS	alexandrin	20.63	-0.2	0.63
PS	Amygdalinic acid	4.15	-2.32	0.63
PS	Benzyl Beta -gentiobioside	3.46	-2.07	0.56
PS	Benzyl glucopyranoside	12.39	-0.7	0.14
PS	Beta-D-Glucopyranoside, 2-((benzoyloxy)methyl)-4-hydroxyphenyl	13.51	-1.03	0.53
PS	Campesterol-3-O-β-D-(6-O-oleyl)glucopyranoside	27.03	0.04	0.17

PS	Campesterol-3-O-β-D-(6-O-palmityl)glucopyranoside	25.65	0.02	0.19
PS	Campesterol-3-O-β-D-glucopyranoside	20.49	-0.22	0.67
PS	Campesterol-3-O-β-D-glucopyranoside_qt	7.86	1.29	0.72
PS	cis-p-Coumarate	45.98	0.46	0.04
PS	D-mandelonitrile	48.26	0.67	0.02
PS	EIC	41.9	1.16	0.14
PS	GA118	10.41	-0.6	0.53
PS	GA121	14.13	-0.26	0.5
PS	GA126	11.8	-0.64	0.53
PS	GA16	14.26	-0.63	0.53
PS	GA30	61.72	-0.83	0.54
PS	GA54	64.21	-0.5	0.53
PS	GA60	93.17	-0.79	0.53
PS	GA61	14.82	-0.22	0.49
PS	GA63	65.54	-0.57	0.54

PS	GA69	17.67	-0.21	0.49
PS	GA70	14.04	-0.23	0.49
PS	GA77	87.89	-0.64	0.53
PS	GA87	68.85	-1.14	0.57
PS	GA95	20.01	-0.24	0.49
PS	GA97	10.12	-0.89	0.47
PS	Gibberellin 17	94.64	-0.87	0.49
PS	Grandidentatin	10.56	-0.9	0.54
PS	Heriguard	11.93	-1.03	0.33
PS	Methyl-alpha-D-fructofuranoside	65.63	-1.09	0.05
PS	MGL	24.46	-1.13	0.05
PS	MNN	48.36	0.68	0.02
PS	Triolein	27.27	0.97	0.13
PS	Prunasin	12.61	-0.79	0.18
PS	RMN	43.67	0.11	0.03

PS	Salireposide_qt	24.3	0.63	0.12
PS	WLN: Q1R	58.68	1.08	0.01
PS	β-sitosterol 3-O-β-D-(6-O-oleyl)glucopyranoside	26.94	-0.02	0.16
PS	β-sitosterol-3-(6-palmitoyl)glucopyranoside	26.07	0.1	0.18
RRR	(-)-Catechin	49.68	-0.03	0.24
RRR	Aloe-emodin	83.38	-0.12	0.24
RRR	Chrysophanol	18.64	0.62	0.21
RRR	Daucosterol {Sitogluside}	20.18	0.09	0.69
RRR	Daucosterol_qt	35.89	1.35	0.7
RRR	Emodin	24.4	0.22	0.24
RRR	Eupatin	50.8	0.53	0.41
RRR	Mutatochrome {Citroxanthin}	48.64	1.97	0.61
RRR	Palmidin A	32.45	-0.36	0.65
RRR	Physcion	22.29	0.52	0.27
RRR	Rhein	47.07	-0.2	0.28

RRR	Toralactone	46.46	0.86	0.24
RRR	Hispidulin	29.95	0.47	0.27
RRR	Procyanidin B2 3,3'-di-O-gallate	42.63	-1.86	
RRR	Palmidin B	1.21	-0.11	0.69
RRR	(-)-Epicatechin-pentaacetate	8	-0.59	0.77
RRR	(+)-Catechin-pentaacetate	27.58	-0.65	0.77
RRR	(9S)-9-[(9R)-2-carboxy-4,5-dihydroxy-10-oxo-9H-anthracen-9-yl]-4,5-dihydroxy-10-oxo-9H-anthracene-2-carboxylic acid	27.75	-0.91	0.57
RRR	[(2R,3R,4S,5R,6R)-3,5-dihydroxy-2-(3,4,5-trihydroxybenzoyl)oxy-6-[(3,4,5-trihydroxybenzoyl)oxymethyl]oxan-4-yl] 3,4,5-trihydroxybenzoate	3.01	-2.22	0.54
RRR	[(2R,3S,4S,5R,6S)-6-[4-[(Z)-2-(3,5-dihydroxyphenyl)ethenyl]phenoxy]-3,4,5-trihydroxyoxan-2-yl]methyl 3,4,5-trihydroxybenzoate	2.69	-1.17	0.73
RRR	[Epicatechin-(48)]5-epicatechin	4.02	-4.48	0
RRR	1,8-dihydroxy-3-methoxy-2,6-dimethyl-9,10-anthraquinone	5.53	0.63	0.29
RRR	10beta-Hydroxy-6beta-isobutyrylfuranoeremophilane	16.94	0.88	0.29
RRR	1-Cinnamoyl-3-hydroxy-11-methoxymeliacarpinin	35.72	-0.58	0.07

RRR	1-O-Galloyl-glycerol	63.21	-0.81	0.1
RRR	1-O-Galloylpedunculagin	38.09	-2.9	0.04
RRR	2,3-Digalloylglucose	2.99	-2.25	0.66
RRR	2-Cinnamoyl-glucose	17.02	-0.73	0.22
RRR	2-Methyl cardol	3.56	1.46	0.28
RRR	2-Methyl-5-carboxymethyl-7-hydroxychromanone	14.8	0.06	0.12
RRR	3,5-Di-O-galloyl-4-O-digalloylquinic acid	3.01	-2.61	0.31
RRR	3-Carboxy-4-hydroxy-phenoxy glucoside	13.85	-1.44	0.22
RRR	3-Hydroxy-25-norfriedel-3,1(10)-dien-2-one-30-oic acid	18.4	0.41	0.78
RRR	5-[(Z)-2-(3-hydroxy-4-methoxy-phenyl)vinyl]resorcinol	76.25	0.66	0.15
RRR	5-acetyl-7-hydroxy-2-methyl-chromone	30.25	0.37	0.1
RRR	5-Carboxy-7-hydroxy-2-methyl-benzopyran-gamma-one	34.4	0.14	0.11
RRR	Aloeemodin-omega-O-beta-D-glucopyranoside	9.04	-1.34	0.81
RRR	Anthraglycoside B	27.06	-1.29	0.8
RRR	Barolub	16.29	0.88	0.22

RRR	Beta-Glucogallin	17.89	-1.58	0.25
RRR	Chrysophanol glucoside	20.06	-1.17	0.76
RRR	Chrysophanol-8-O-beta-D-(6'-O-galloyl)-glucopyranoside	1.92	-1.48	0.69
RRR	Cinnamic acid	19.68	0.91	0.03
RRR	cis-Zimtsaeure	38.19	0.91	0.03
RRR	Citric acid	56.22	-1.11	0.05
RRR	DLA	44.51	-0.21	0.01
RRR	DMR	68.62	-1.09	0.02
RRR	Docosanoate	15.69	1.21	0.26
RRR	Emodin-1-O-beta-D-glucopyranoside	44.81	-1.12	0.8
RRR	Emodin-6-glucoside	16.09	-1.28	0.8
RRR	Emodinanthrone	24.72	0.8	0.21
RRR	Gallic acid-3-O-(6'-O-galloyl)-glucoside	30.25	-1.96	0.67
RRR	Gallic acid-4-O-(6'-O-galloyl)-glucoside	27.06	-1.82	0.67
RRR	Gallocatechin	2.26	-0.27	0.27

RRR	Glycerite	7.28	-4.71	0.03
RRR	Laccaic acid D	2.06	-0.56	0.31
RRR	MAE	65.06	-0.46	0.01
RRR	o-Acetyltoluene	38.96	1.47	0.02
RRR	OXL	29.68	-0.64	0.01
RRR	Palmidin C	2.35	0.18	0.69
RRR	Physcion-8-O-beta-D-glucopyranoside	8.2	-1.19	0.85
RRR	Physcion-9-O-beta-D-glucopyranoside_qt	20.3	0.56	0.3
RRR	Physciondiglucoside	41.65	-2.64	0.63
RRR	Physcione	19.38	0.02	0.27
RRR	PIT	72.29	0.64	0.13
RRR	Procyanidin B-5,3'-O-gallate {Procyanidin}	31.99	-1.61	0.32
RRR	RHAPONTIN	9	-0.86	0.55
RRR	Rhapontisterone	4.39	-1.84	0.82
RRR	Rheidin B	1.21	-0.19	0.65

RRR	Rheidin C	1.24	-0.18	0.58
RRR	Rhein diglucoside	2.93	-2.79	0.63
RRR	Rheinoside A	0.82	-3.17	0.68
RRR	Rheinoside A_qt	26.28	-1.87	0.75
RRR	Rheochrysin	18.31	-1.14	0.82
RRR	Rheosmin	26.79	0.97	0.04
RRR	Rheumin	9.16	-0.23	0.17
RRR	Sennidin C	1.28	-0.62	0.61
RRR	Sennoside A	3.34	-3.7	0.08
RRR	Sennoside B	3.34	-3.85	0.08
RRR	Sennoside C	3.99	-3.54	0.09
RRR	Sennoside D	3.99	-3.48	0.09
RRR	Sennoside D_qt	61.06	-0.7	0.61
RRR	Sennoside E	3.02	-3.67	0.06
RRR	Sennoside E_qt	50.69	-0.74	0.61

RRR	Serotonin	42.99	0.59	0.06
RRR	Strumaroside	20.78	-0.23	0.67
RRR	Succinic acid	29.62	-0.44	0.01
RRR	Torachrysone-8-O-beta-D-(6'-oxayl)-glucoside	43.02	-1.23	0.74
RRR	TPBO	24.23	1.42	0.03
RRR	ZINC04081604	15.23	0.88	0.44
MRC/RRR	3,4,5-trihydroxybenzoic acid {Gallic acid}	31.69	-0.09	0.04
PS/RRR	β-Sitosterol	36.91	1.32	0.75

MRC, Moutan Radicis Cortex; PS, Persicae Semen; RRR, Rhei Radix et Rhizoma; OB, oral bioavailability; Caco-2, Caco-2 cell permeability; DL, drug-likeness score. Synonyms of certain chemical compounds are given in braces {}.

Supplementary Table S3. Active chemical components of FDY2004.

Herbal medicines	Chemical compounds	OB	Caco-2	DL
MRC	(+)-Catechin	54.83	-0.03	0.24
MRC	5-[[5-(4-methoxyphenyl)-2-furyl]methylene]barbituric acid {ZINC9320615}	43.44	0.09	0.3
MRC	Caffeic acid	25.76	0.21	0.05
MRC	Kaempferol	41.88	0.26	0.24
MRC	Mairin	55.38	0.73	0.78
MRC	Paeoniflorin	10.22	-1.69	0.79
MRC	Paeonol	28.79	0.93	0.04
MRC	Pentagalloylglucose	3.01	-3.08	0.21
MRC	Quercetin	46.43	0.05	0.28
MRC	Sitosterol	36.91	1.32	0.75
PS	(+)-Epicatechin	48.96	0.02	0.24
PS	Amygdalin	4.42	-1.91	0.61

PS	Campesterol	37.58	1.31	0.71
PS	GA120	84.85	0.38	0.45
PS	GA121-Isolactone	72.7	-0.26	0.54
PS	GA122	64.79	-0.17	0.5
PS	GA122-Isolactone	88.11	-0.18	0.54
PS	Gibberellin A119	76.36	-0.12	0.49
PS	Gibberellin A44	101.61	-0.13	0.54
PS	Gibberellin A7	73.8	-0.18	0.5
PS	Hederagenin	36.91	1.32	0.75
PS	α1-Sitosterol	43.28	1.41	0.78
RRR	(-)-Catechin	49.68	-0.03	0.24
RRR	Aloe-emodin	83.38	-0.12	0.24
RRR	Chrysophanol	18.64	0.62	0.21
RRR	Daucosterol {Sitogluside}	20.18	0.09	0.69
RRR	Emodin	24.4	0.22	0.24

RRR	Eupatin	50.8	0.53	0.41
RRR	Mutatochrome {Citroxanthin}	48.64	1.97	0.61
RRR	Palmidin A	32.45	-0.36	0.65
RRR	Physcion	22.29	0.52	0.27
RRR	Rhein	47.07	-0.2	0.28
RRR	Toralactone	46.46	0.86	0.24
MRC/RRR	3,4,5-trihydroxybenzoic acid {Gallic acid}	31.69	-0.09	0.04
PS/RRR	β-Sitosterol	36.91	1.32	0.75

MRC, Moutan Radicis Cortex; PS, Persicae Semen; RRR, Rhei Radix et Rhizoma; OB, oral bioavailability; Caco-2, Caco-2 cell permeability; DL, drug-likeness score. Synonyms of certain chemical compounds are given in braces {}.

Supplementary Table S4. Targets of active chemical components of FDY2004.

Herbal medicines	Chemical compounds	Targets
MRC	(+)-Catechin	ALPL*, APOB*, BACE1*, CA1, CA2, CA3, CA4*, CA5A, CA5B, CA6, CA7, CA9*, CA12*, CSF2*, DNMT1*, FUT4*, HMOX1*, KIAA1149, PGD*, PGF*, PON1*, PTGS2*, SLC22A11, SLC47A1
MRC	Caffeic acid	ALOX5*, CA1, CA2, CA3, CA5A, CA5B, CA6, CA7, CA9*, CA12*, CA14, MMP1*, MMP2*, MMP9*, NR0B2*, PTPN1*, SLC22A6
MRC	Kaempferol	ABCB1*, ABCC1*, ABCG2*, AHR*, AKR1B1*, ALOX5*, AR*, CA2, CA7, CA12*, CDK1*, CISD1, CTDSP1, CYP1A2*, CYP1B1*, CYP2D6*, DAPK1, ESRRA*, FLT3*, HSD17B1*, HSD17B2*, MPO*, NOX4*, NR1I2*, P4HB, PTPRS, RPS6KA3*, SLC2A1*, TYR*, UGT1A3, UGT1A7*, UGT1A8, UGT1A9*, UGT3A1, XDH*
MRC	Mairin	A1CF, AKR1B10*, AMD1, ARHGEF7*, CDCA8*, CPSF4*, CYP2R1, DHPS, EIF2AK2*, EME1, FOLH1*, GPBAR1, HIGD1B, MDM2*, MEX3D, MMAB, MTOR*, POLB*, PTPRN, RAC1*, RNF31, RPS3, S100A8*, SAE1, UBA2*, USF1
MRC	Mutatochrome {Citroxanthin}	DRAP1, NR1D2*, RANBP2, RUVBL1*, TJP1*, TPK1, YAP1*
MRC	Paeoniflorin	BLVRA, F13A1*, FRS3, HNRNPR, NUCB1, STRBP*
MRC	Paeonol	CASP8*, CASP10*, ICAM1*, MTNR1A*, MTNR1B, PTGS2*, TYR*, VCAM1*

MRC	Pentagalloylglucose	ABCC11*, AMY1A, AMY1B, AMY1C, BACE1*, EGFR*, ESR1*, F10*, HMOX1*, MCL1, NFE2L2*, PTPN1*, PTPN2*, SERPINE1*, SQLE*, TMPRSS11D
MRC	Quercetin	ABCB1*, ABCC1*, ABCG2*, AKR1B1*, AKT1*, ALK*, ALOX12*, ALOX15*, ALOX5*, ATP5B, AURKB*, AVPR2*, AXL*, BACE1*, CA1, CA2, CA3, CA4*, CA5A, CA6, CA7, CA9*, CA12*, CA14, CAMK2B, CCR4*, CDK1*, CSNK2A1*, CXCR1*, CYP1A1*, CYP1A2*, CYP1B1*, CYP2C8*, CYP2C9*, DAPK1, DRD4*, EGFR*, ELAVL1*, F2*, FLT3*, GLO1, GPR35, GSK3B*, HCK, HIBCH, HSD17B2*, IGF1R*, KDR*, MAOA*, MCL1, MET*, MMP2*, MMP3*, MMP9*, MMP13*, MPO*, NEK2*, NEK6, NOX4*, NUAK1, P4HB*, PIK3R1, PIM1*, PKN1, PLA2G1B*, PTK2*, PTPRS, PYGL, SLC2A2, STK17B, XDH*
MRC	Sitosterol	CALML3, CYP17A1, DHPS, DRAP1, GPBAR1, HDLBP, LTF*, NPC1L1, NR1D2*, RAC1*, RUVBL1*, USF1
PS	(+)-Epicatechin	ALPL, APOB, BACE1, CA1, CA2, CA3, CA4, CA5A, CA5B, CA6, CA7, CA9, CA12, CSF2, DNMT1, FUT4, HMOX1, KIAA1149, PGD*, PGF*, PON1*, PTGS2*, SLC22A11, SLC47A1
PS	Campesterol	AMD1, ARHGEF7, CALML3, CDCA8, CYP17A1, DHPS, DRAP1, EIF2AK2, GPBAR1, LTF*, MDM2*, MEX3D, MTOR*, NPC1L1, NR1D2*, PTPRN, RAC1*, RPS3, S100A8*, SHBG*, SLC10A1, SRD5A2*, TNNC1, USF1, USP8*
PS	GA120	ACBD7, AMD1, CALML3, EIF2AK2, MDM2*, MEX3D, MTOR*, RAC1*, RPS3, S100A8*

PS	GA121-Isolactone	ARHGEF7, CPSF4, CYP2R1, DHRS1, EIF2AK2, FOLH1, MDM2*, MEX3D, MMAB, MTOR*, MTRR*, PHO85, PTPRN, RAC1*, RGS18, RPS3
PS	GA122-Isolactone	DHRS1, MTRR*, RGS18
PS	Gibberellin A44	BRD7, DHRS1
PS	Gibberellin A7	ACBD7, EIF2AK2*, MDM2*, MEX3D, MTOR*, RAC1*
PS	Hederagenin	F3*, PNPN1, PTPN2*
PS	α1-Sitosterol	ACBD7, AMD1, CALML3*, CDCA8*, DHPS, DRAP1, EIF2AK2*, HDLBP, LTF*, MDM2*, MEX3D, MTOR*, NR1D2*, RAC1*, RANBP2, RNF31, RUVBL1*, TNNC1, USF1, USP8*
RRR	(-)-Catechin	ALPL*, APOB*, BACE1*, CA1, CA2, CA3, CA4*, CA5A, CA5B, CA6, CA7, CA9*, CA12*, CSF2*, DNMT1*, FUT4*, HMOX1*, KIAA1149, PGD*, PGF*, PON1*, PTGS2*, SLC22A11, SLC47A1
RRR	Aloe-emodin	CASP3*, CASP8*, CASP9*, EIF2S1, HSP90AA1*, TP53*, UGT1A9*, XBP1*
RRR	Chrysophanol	NLRP3*, UGT1A9*
RRR	Daucosterol {Sitogluside}	CYP17A1*, NPC1L1, NR1D2*, RUVBL1*
RRR	Emodin	AKR1B1*, CASP3*, CSNK2A1*, CSNK2B, CXCR4*, CYP1B1*, ERBB2*, ESR1*, ESR2*, LCK*, MCL1, PIM1*, PTP4A3*,TNF*, TP53*, VEGFA*

RRR	Eupatin	CREB1*, CYP1B1*
RRR	Physcion	ELANE*, UGT1A9*
RRR	Rhein	DNM1L*, FTO*, NFKB1*, PPARA*, PPARG*, RELA*, RXRA*, SLC37A4, TPMT*, UCP1, VEGFA*
MRC/RRR	3,4,5-trihydroxybenzoic acid {Gallic acid}	ABCB1*, ATM*, CA1, CA2, CA3, CA4*, CA5A, CA5B, CA6, CA7, CA9*, CA12*, CA14, CASP3*, EIF2AK3*, FUT7*, GATA3*, JUN*, MMP2*, SERPINE1*, TPMT*, TYR*, UGT2B17*
PS/RRR	β-Sitosterol	ACBD7, CYP17A1*, DHPS, DRAP1, GPBAR1, LTF*, MDM2*, MEX3D, MTOR*, NPC1L1, NR1D2, RAC1*, RANBP2, RUVBL1*, TNNC1, USF1

MRC, Moutan Radicis Cortex; PS, Persicae Semen; RRR, Rhei Radix et Rhizoma; *, lung cancer-associated targets. Synonyms of certain chemical compounds are given in braces {}.

Supplementary Table S5. Docking scores between active chemical components of FDY2004 and the lung cancer-associated targets.

Chemical compounds	Targets											
	AKT1	EGFR	ESR1	HSP90AA1	JUN	NFKB1	PIK3R1	PTK2	RAC1	TNF	TP53	VEGFA
(-)-Catechin	-5.7	-7.7	-7.0	-6.7	-5.3	-6.3	-6.1	-6.0	-8.5	-6.2	-7.3	-6.9
(+)-Catechin	-6.0	-7.7	-7.0	-6.7	-5.3	-6.3	-6.1	-6.0	-9.0	-6.2	-7.3	-6.9
(+)-Epicatechin	-5.6	-7.7	-7.0	-6.7	-5.3	-6.4	-6.1	-6.0	-9.0	-6.3	-7.3	-6.9
Aloe-emodin	-6.1	-8.3	-7.4	-7.2	-5.4	-6.4	-6.0	-6.1	-9.6	-6.4	-7.1	-6.8
Caffeic acid	-5.2	-6.5	-5.1	-5.8	-4.6	-5.3	-5.0	-4.8	-6.8	-4.9	-5.8	-5.5
Campesterol	-6.0	-9.4	-6.4	-5.7	-5.8	-5.8	-6.0	-5.9	-8.3	-5.6	-6.2	-7.9
Chrysophanol	-5.7	-8.5	-7.6	-7.3	-5.4	-6.5	-5.8	-6.0	-9.8	-6.7	-7.3	-7.0
Daucosterol	-5.3	-7.2	-8.1	-5.1	-5.8	-6.9	-6.0	-6.7	-8.8	-5.9	-6.6	-7.9
Emodin	-6.0	-8.3	-7.8	-7.2	-5.6	-6.5	-5.8	-6.2	-10.1	-6.8	-7.3	-7.0
Eupatin	-5.5	-8.7	-6.8	-6.0	-5.4	-6.8	-5.7	-6.0	-9.3	-6.7	-6.6	-7.1
GA120	-6.9	-8.2	-7.3	-7.4	-6.7	-6.5	-6.7	-6.2	-8.4	-6.1	-7.1	-7.5
Gallic acid	-5.4	-6.4	-5.4	-5.2	-5.1	-5.2	-4.9	-4.6	-6.3	-4.8	-5.4	-5.6

Gibberellin A7	-6.0	-7.9	-7.0	-7.2	-6.5	-6.4	-6.5	-6.2	-8.1	-6.4	-6.9	-7.4
Hederagenin	-6.5	-10.0	-7.9	-5.0	-6.6	-6.5	-7.5	-6.9	-7.7	-6.5	-7.4	-7.8
Kaempferol	-6.3	-7.9	-6.6	-6.3	-5.4	-6.4	-6.1	-5.7	-8.8	-6.3	-6.7	-6.9
Mairin	-5.0	-9.8	-7.9	-6.5	-6.8	-6.5	-6.8	-6.7	-6.8	-6.6	-7.8	-8.3
Mutatochrome	-6.9	-5.4	-7.3	-6.5	-7.3	-7.7	-7.8	-7.2	-10.3	-6.2	-7.6	-9.6
Paeoniflorin	-6.0	-8.6	-7.0	-6.1	-6.1	-6.8	-6.7	-7.2	-8.4	-5.7	-6.6	-7.6
Paeonol	-5.3	-5.7	-5.0	-5.4	-3.9	-4.9	-4.5	-4.1	-6.3	-4.9	-5.2	-5.1
Pentagalloylglucose	-6.0	-8.9	-7.0	-3.0	-6.0	-8.7	-6.3	-7.0	-8.9	-7.0	-8.3	-8.4
Physcion	-8.1	-11.2	-9.9	-6.3	-7.8	-8.8	-8.0	-9.1	-12.7	-8.3	-8.8	-10.4
Quercetin	-6.4	-7.9	-6.5	-6.1	-5.4	-6.6	-6.3	-6.1	-9.3	-6.5	-7.0	-7.2
Rhein	-5.4	-8.7	-7.6	-6.7	-5.6	-6.8	-6.1	-6.2	-10.2	-6.4	-7.3	-7.1
Sitosterol	-4.5	-8.8	-6.2	-5.5	-5.8	-5.3	-5.9	-5.9	-8.1	-5.6	-6.4	-7.8
α1-Sitosterol	-4.8	-9.7	-6.3	-6.8	-6.3	-5.5	-5.9	-5.9	-7.8	-5.5	-7.0	-8.5
-Sitosterol	-4.5	-8.8	-6.2	-5.2	-5.9	-5.3	-5.8	-5.8	-7.0	-5.6	-6.8	-7.9

References

1. M. Zhang, L. Feng, J. Gu, et al. "The attenuation of Moutan Cortex on oxidative stress for renal injury in AGEs-induced mesangial cell dysfunction and streptozotocin-induced diabetic nephropathy rats," *Oxid Med Cell Longev, vol.* 2014, p. 463815.

2. H.S. Choi, H.S. Seo, J.H. Kim, J.Y. Um, Y.C. Shin, and S.G. Ko. "Ethanol extract of paeonia suffruticosa Andrews (PSE) induced AGS human gastric cancer cell apoptosis via fas-dependent apoptosis and MDM2-p53 pathways," *J Biomed Sci, vol.* 19, p. 82.

3. H.S. Kim, A.R. Kim, J.M. Lee, et al. "A mixture of Trachelospermi caulis and Moutan cortex radicis extracts suppresses collagen-induced arthritis in mice by inhibiting NF-kappaB and AP-1," *J Pharm Pharmacol, vol.* 64, no. 3, pp. 420-429.

4. S.Y. Zong, Y.Q. Pu, B.L. Xu, T. Zhang, and B. Wang. "Study on the physicochemical properties and anti-inflammatory effects of paeonol in rats with TNBS-induced ulcerative colitis," *Int Immunopharmacol, vol.* 42, pp. 32-38.

5. W. Jia, X. Wang, D. Xu, A. Zhao, and Y. Zhang. "Common traditional Chinese medicinal herbs for dysmenorrhea," *Phytother Res, vol.* 20, no. 10, pp. 819-824.

6. H.Y. Chen, Y.H. Lin, I.H. Su, Y.C. Chen, S.H. Yang, and J.L. Chen. "Investigation on Chinese herbal medicine for primary dysmenorrhea: implication from a nationwide prescription database in Taiwan," *Complement Ther Med, vol.* 22, no. 1, pp. 116-125.

7. H.J. Kim, K.K. Park, W.Y. Chung, S.K. Lee, and K.R. Kim. "Protective Effect of White-fleshed Peach (Prunus persica (L.) Batsch) on Chronic Nicotine-induced Toxicity," *J Cancer Prev, vol.* 22, no. 1, pp. 22-32.

8. C. Burks-Wicks, M. Cohen, J. Fallbacher, N.T. R, and F. Wieser. "A Western primer of Chinese herbal therapy in endometriosis and infertility," *Womens Health (Lond), vol.* 1, no. 3, pp. 447-463.

9. Y.C. Wu, P. Wu, Y.B. Li, T.C. Liu, L. Zhang, and Y.H.J.R.a. Zhou. "Natural deep eutectic solvents as new green solvents to extract anthraquinones from Rheum palmatum L," *RSC Adv, vol.* 8, no. 27, pp. 15069-15077.

10. Y. Fan, Z. Niu, C. Xu, L. Yang, and T. Yang. "Protic Ionic Liquids as Efficient Solvents in Microwave-Assisted Extraction of Rhein and Emodin from Rheum palmatum L," *Molecules, vol.* 24, no. 15.

11. I.-H. Lee, H.-S. Lee, K. Kang, et al. "Influence of Decoction Duration of FDY2004 on Its Physicochemical Components and Antioxidant and Antiproliferative Activities," *Nat Prod Commun, vol.* 15, no. 10, p. 1934578X20968437.

12. M.H. Zhang, L. Feng, M.M. Zhu, et al. "The anti-inflammation effect of Moutan Cortex on advanced glycation end products-induced rat mesangial cells dysfunction and High-glucose-fat diet and streptozotocin-induced diabetic nephropathy rats," *J Ethnopharmacol, vol.* 151, no. 1, pp. 591-600.

13. P.K. Fu, C.Y. Yang, T.H. Tsai, and C.L. Hsieh. "Moutan cortex radicis improves lipopolysaccharide-induced acute lung injury in rats through anti-inflammation," *Phytomedicine, vol.* 19, no. 13, pp. 1206-1215.

14. Z. Wang, C. He, Y. Peng, F. Chen, and P. Xiao. "Origins, Phytochemistry, Pharmacology, Analytical Methods and Safety of Cortex Moutan (Paeonia suffruticosa Andrew): A Systematic Review," *Molecules, vol.* 22, no. 6.

15. G.J. Kim, H.G. Choi, J.H. Kim, S.H. Kim, J.A. Kim, and S.H. Lee. "Anti-allergic inflammatory effects of cyanogenic and phenolic glycosides from the seed of Prunus persica," *Nat Prod Commun, vol.* 8, no. 12, pp. 1739-1740.

16. K.H. Seo, S.Y. Choi, Y. Jin, et al. "Antiinflammatory role of Prunus persica L. Batsch methanol extract on lipopolysaccharidestimulated glial cells," *Mol Med Rep, vol.* 21, no. 5, pp. 2030-2040.

17. W. Cassiem and M. de Kock. "The anti-proliferative effect of apricot and peach kernel extracts on human colon cancer cells in vitro," *BMC Complement Altern Med, vol.* 19, no. 1, p. 32.

18. T. Fukuda, H. Ito, T. Mukainaka, H. Tokuda, H. Nishino, and T. Yoshida. "Anti-tumor promoting effect of glycosides from Prunus persica seeds," *Biol Pharm Bull, vol.* 26, no. 2, pp. 271-273.

19. R. Kant, R.K. Shukla, A.J.I.J.F.R.I.A.S. Shukla, and E. Technology. "A review on Peach (Prunus persica): an asset of medicinal phytochemicals," *Int J Res Appl Sci Eng Technol, vol.* 6, no. I.

20. S. Kim, S.Y. Hong, B. Kwon, et al. "Effect of Persicae Semen for Atopic Dermatitis Skin Tissue and Regulate to Inflammation Mediator in Serum," *Korea J Herbol, vol.* 35, no. 4, pp. 51-60.

21. C.S. Shia, S.H. Juang, S.Y. Tsai, et al. "Metabolism and pharmacokinetics of anthraquinones in Rheum palmatum in rats and ex vivo antioxidant activity," *Planta Med, vol.* 75, no. 13, pp. 1386-1392.

22. Z.B. Tan, H.J. Fan, Y.T. Wu, et al. "Rheum palmatum extract exerts anti-hepatocellular carcinoma effects by inhibiting signal transducer and activator of transcription 3 signaling," *J Ethnopharmacol, vol.* 232, pp. 62-72.

23. H. Xiang, J. Zuo, F. Guo, and D. Dong. "What we already know about rhubarb: a comprehensive review," *Chin Med, vol.* 15, p. 88.
